# Spinal Cord Stimulation for the Treatment of Refractory Pain From Tarlov Cysts: A Case Report

**DOI:** 10.7759/cureus.33928

**Published:** 2023-01-18

**Authors:** Jungmin On, Javier J Polania Gutierrez, Miguel Plaza-Lloret, Anterpreet Dua, Zhuo Sun

**Affiliations:** 1 Anesthesiology, Rush University Medical Center, Chicago, USA; 2 Anesthesiology, Augusta University Medical College of Georgia, Augusta, USA

**Keywords:** interventional pain medicine, chronic low back pain (clbp), tarlov cyst, spinal cord stimulation (scs), chronic pain management

## Abstract

Tarlov cysts are extradural meningeal cysts with a collection of cerebrospinal fluid that most often affects sacral nerve roots, causing chronic low back pain and radiculopathy. Still, there is no consensus regarding the best treatment for symptomatic cysts. We describe a patient who developed worsening lower back pain and radiculopathy after interventional drainage and surgical management. Medication and various procedures failed to relieve the pain. Subsequently, the patient received significant pain relief from spinal cord stimulation (SCS). This case provides evidence that SCS could be used to manage refractory pain from Tarlov cysts that have failed to respond to other treatment modalities.

## Introduction

Tarlov cysts are rare extradural meningeal cysts that were first described in 1938 as incidental findings on autopsy. They are perineural CSF-filled sacs that usually arise from the lumbosacral nerve roots near the dorsal root ganglion [1]. The wall of the cyst is continuous with the arachnoid mater and dura mater of the posterior roots and its cavity is contained between the perineurium and endoneurium. The prevalence of these cysts in the general population has been estimated to be between 1.5% and 4.6%, and less than 1% of cysts cause symptoms depending on the location within the spinal canal. While often asymptomatic, these cysts may grow in size and manifest with a range of neurologic symptoms, including chronic low-back pain, sacrococcygeal pain, perineal pain, radiculopathy, changes in bladder and bowel function, and sexual dysfunction [2]. The diagnosis is usually made using lumbosacral or pelvic magnetic resonance imaging (MRI).

While several treatment modalities have been described in the literature for symptomatic Tarlov cysts, there has been wide variability in symptom resolution, cyst recurrence, and post-procedural complications. Non-surgical strategies include minimally invasive techniques such as CT- or fluoroscopy-guided aspiration and percutaneous fibrin glue injection [2,3]. Surgical techniques include decompressive laminectomy, cyst cauterization, cyst excision, cyst resection, cyst shunting, and neck ligation together with duraplasty or plication of the cyst wall. Each of these procedures is associated with multiple complications including pseudomeningocele, hemorrhage, intracranial hypotension, neurological deficit, CSF leakage, infection, CSF fistula, aseptic meningitis, and allergic reactions to sealants, with a high risk of cyst recurrence. A 2019 meta-analysis by Sharma et al. demonstrated a complication rate of 21% for surgical management and 12.47% for non-surgical management of Tarlov cysts, respectively, yet 83.5% of patients experienced a symptomatic improvement in both groups. Though surgery was associated with a higher complication rate, the long-term efficacy and success of symptom resolution were superior following surgery rather than percutaneous techniques [4].

Spinal cord stimulation (SCS) was first described almost 50 years ago and its application in the management of chronic pain has developed rapidly since its advent. There is strong evidence of the safety, efficacy, and cost-effectiveness of SCS in the treatment of chronic back pain in particular, with more than half of all patients experiencing significant, sustained pain reduction following treatment [5]. The successful use of SCS in the management of a symptomatic Tarlov cyst was previously described by Hasoon et al. in a 66-year-old woman with worsening pelvic pain following non-surgical and surgical management [6]. Here, we describe a patient with a Tarlov cyst with chronic back pain and lumbar radiculopathy refractory to treatment, who also received significant symptomatic improvement following SCS implantation.

## Case presentation

**Figure 1:**
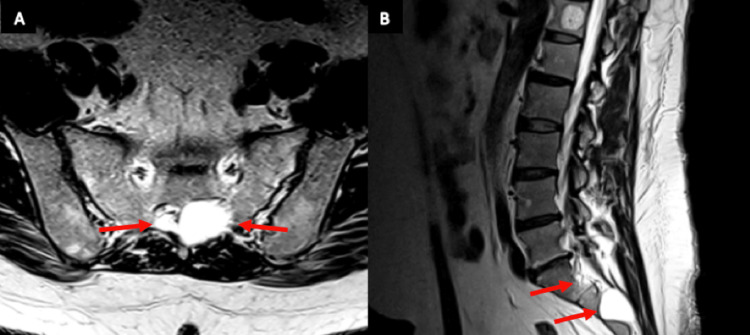
MRI views of Tarlov cysts affecting sacral nerve roots A: Axial MRI view. The red arrows identify multiple type II meningeal (Tarlov) cysts within the sacral foramina; B: Sagittal MRI view. The red arrows identify multiple type II meningeal (Tarlov) cysts at the level of S1-S2

The patient was a 43-year-old man with a longstanding history of chronic low back pain secondary to multiple Tarlov cysts affecting the L1-S2 nerve roots (Figure 1). Symptoms included lower back pain with radiculopathy affecting the bilateral lower extremities, with associated numbness and weakness. Initially, the pain was well-controlled with medication alone, including nonsteroidal anti-inflammatory drugs (NSAIDs), neuropathic pain medicines, and opioids. This regimen was effective for approximately 10 years but then required dosage increments to achieve therapeutic effects progressively increased due to the worsening of pain. 

The patient sought consultation and received multiple rounds of epidural steroid injections, which provided relief for days to weeks. He then sought surgical management and underwent an L4-L5 lumbar discectomy, which was well-tolerated but did not provide any pain relief. Aspiration of the cyst was then attempted, which provided relief for just two days before the return and worsening of low back pain and radiculopathy. Multiple rounds of aspiration were completed in the following months, yet pain relief was only achieved for a day following each attempt. The pain and radiculopathy continued to worsen, and the patient developed issues with urinary frequency and hesitancy.

**Figure 2:**
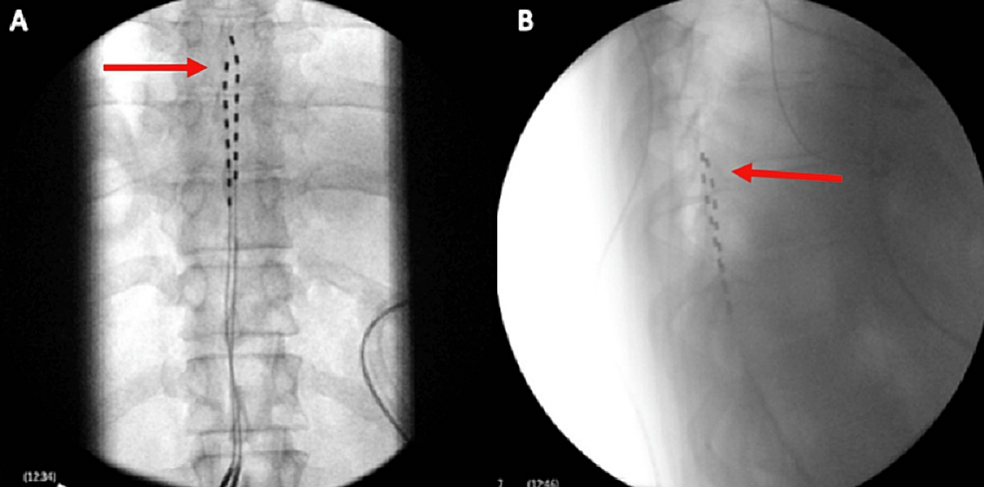
Fluoroscopic views of spinal cord stimulator leads A: Fluoroscopic anterior-posterior view of the spine. The red arrow identifies the tip of the stimulator leads at the superior border of the T8 vertebral body; B: Fluoroscopic lateral view of the spine. The red arrow identifies the stimulator leads in the epidural space and the tip of the leads at the superior border of the T8 vertebral body.

As the patient did not respond favorably to the above-mentioned modalities, a spinal cord stimulation trial was attempted. After discussing the risk and benefits of this treatment, patient consent was obtained. The SCS Medtronic device (Minneapolis, Minnesota) was used and programmed to traditional paresthesia therapy. At the end of the SCS trial, the patient reported a 70-80% reduction in pain and expressed the desire to proceed with the permanent implantation of the SCS Medtronic device. The patient underwent an uneventful SCS implantation. The tips of the leads were positioned at the superior endplate and midbody of the T8 vertebra (Figure 2). The patient presented for a follow-up 15 days after the procedure and reported a 60-70% reduction in pain. At the one-month and eight-month follow-ups, the patient reported a 70-80% reduction in pain and expressed satisfaction with the results.

## Discussion

There is currently no consensus regarding the best treatment modality for symptomatic Tarlov cysts. Patients have historically found significant pain relief from long-term medication use, as well as both non-surgical and surgical management, yet the expanding nature of these cysts and their propensity for recurrence makes their treatment a challenging endeavor. Additionally, each of these modalities is associated with a wide range of adverse effects or complications.

Patients presenting with symptomatic Tarlov cysts, such as those with chronic lumbosacral pain, radiculopathy, and limb weakness, should first undergo conservative management, including NSAIDs, neuropathic medications, and, finally, opioids. If symptoms persist despite medical management, non-surgical management, such as epidural steroid injections and CT-guided aspiration, should be attempted. Surgical intervention may be pursued if other measures fail to relieve pain but should not be the initial treatment due to relatively high complication rates [7].

Spinal meningeal cysts can be classified according to a system [8]. Type I is defined as an extradural meningeal cyst without neural tissue, including an extradural spinal arachnoid cyst (type 1a) and a sacral meningocele (type Ib). A Tarlov cyst is a type-II meningeal cyst, defined as a cyst formed within the nerve-root sheath at the dorsal root ganglion [9]. Most frequently, Tarlov cysts are located in the spinal canal of the S1-to-S5 region of the spinal cord, much less often in the cervical, thoracic or lumbar spine. Type III is defined as an intradural spinal arachnoid cyst. The thoracic and lumbar MRI was reviewed before the procedure, in which no significant spinal canal stenosis was found. MRI did find type II meningeal cysts within both the C7-T1 neural foramina, left T1-T2 neural foramen, both T2-T3 neural foramina, right T6-T7 neural foramen, right T7-T8 neural foramen, and right T10-T11 neural foramen. These findings are consistent with the concept that Tarlov cysts are formed within the dorsal root ganglion. SCS should be a safe procedure to be performed for Tarlov cysts patients.

SCS has been shown to be effective in the management of a variety of types of chronic pain, and there is growing evidence of improvement in both safety and cost-effectiveness [10,11]. However, the application of SCS on symptomatic Tarlov cysts is very rare. The only case report we found is to treat Tarlov cyst-induced pelvic pain [6]. In this case report, we demonstrated the utility of SCS in the management of chronic low back pain and radiculopathy secondary to the Tarlov cyst that had been refractory to a wide range of interventions. This provides further evidence that SCS may be an effective treatment modality for symptomatic cysts that have failed with other measures.

## Conclusions

Tarlov cysts are rare findings with an estimated prevalence of 1.5%-4.6% in the general population, with less than 1% of cysts causing symptoms, including chronic pain and a wide range of neurologic issues. There is no current consensus on the best strategy for the treatment of these cysts, but both conservative management and surgical intervention have been attempted with varying degrees of success. While surgical management is associated with better long-term resolution of symptoms, it also has a higher incidence of complications. This case report provides evidence that spinal cord stimulation may be an equally effective modality in the management of symptomatic Tarlov cysts that are refractory to other treatments.
